# Supplementary figures and images for: Genome-Wide Variation Analysis of Four Vegetable Soybean Cultivars Based on Re-Sequencing
Source: Plants (Basel). 2021 Dec 23;11(1):28. doi: 10.3390/plants11010028 (PMC8747356; doi:10.3390/plants11010028)

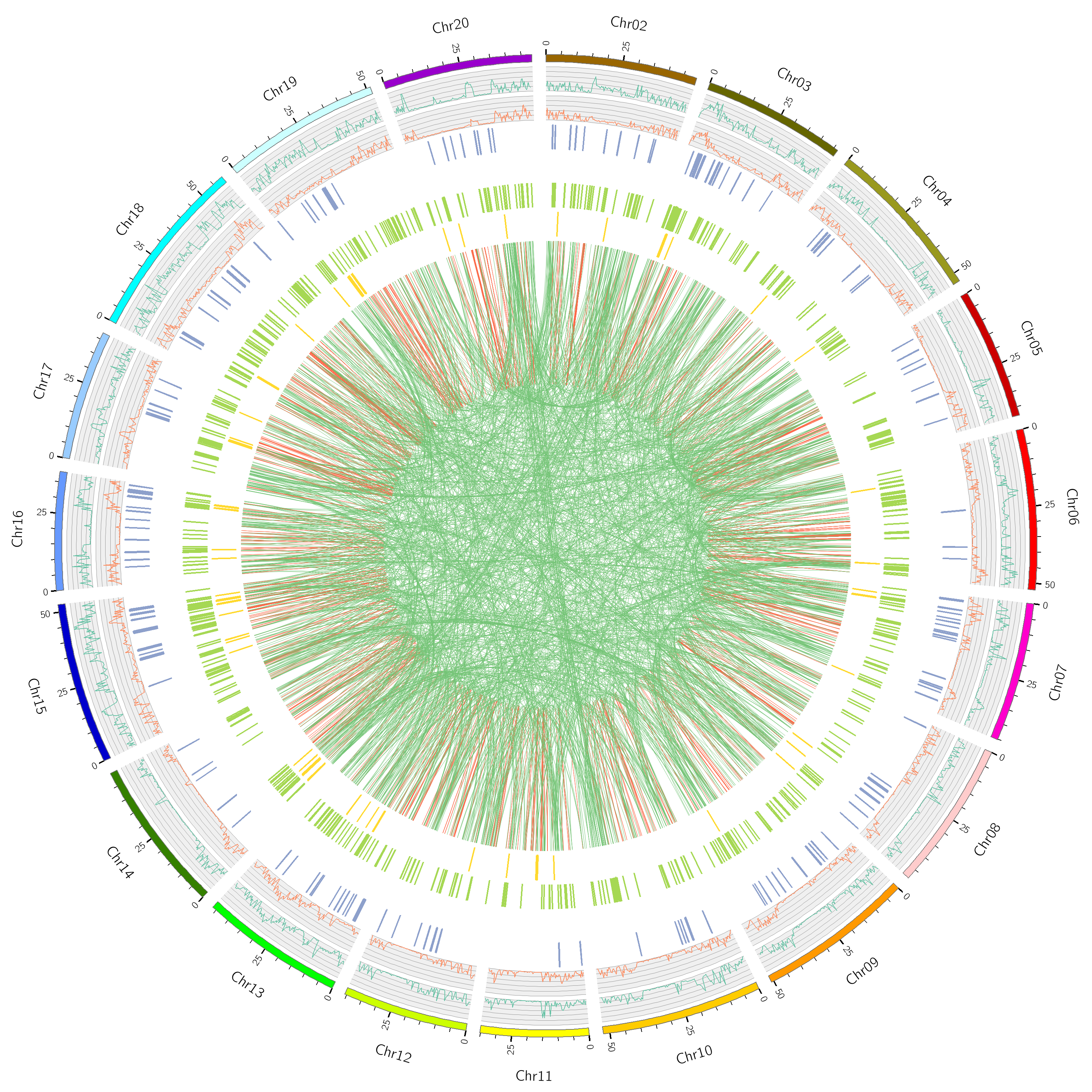

Supplement: Supplementary file 1 [file plants-11-00028-s001.zip › Figure S1. The distribution of the variation of Zhexiandou No. 8 on the chromosomes.jpg]

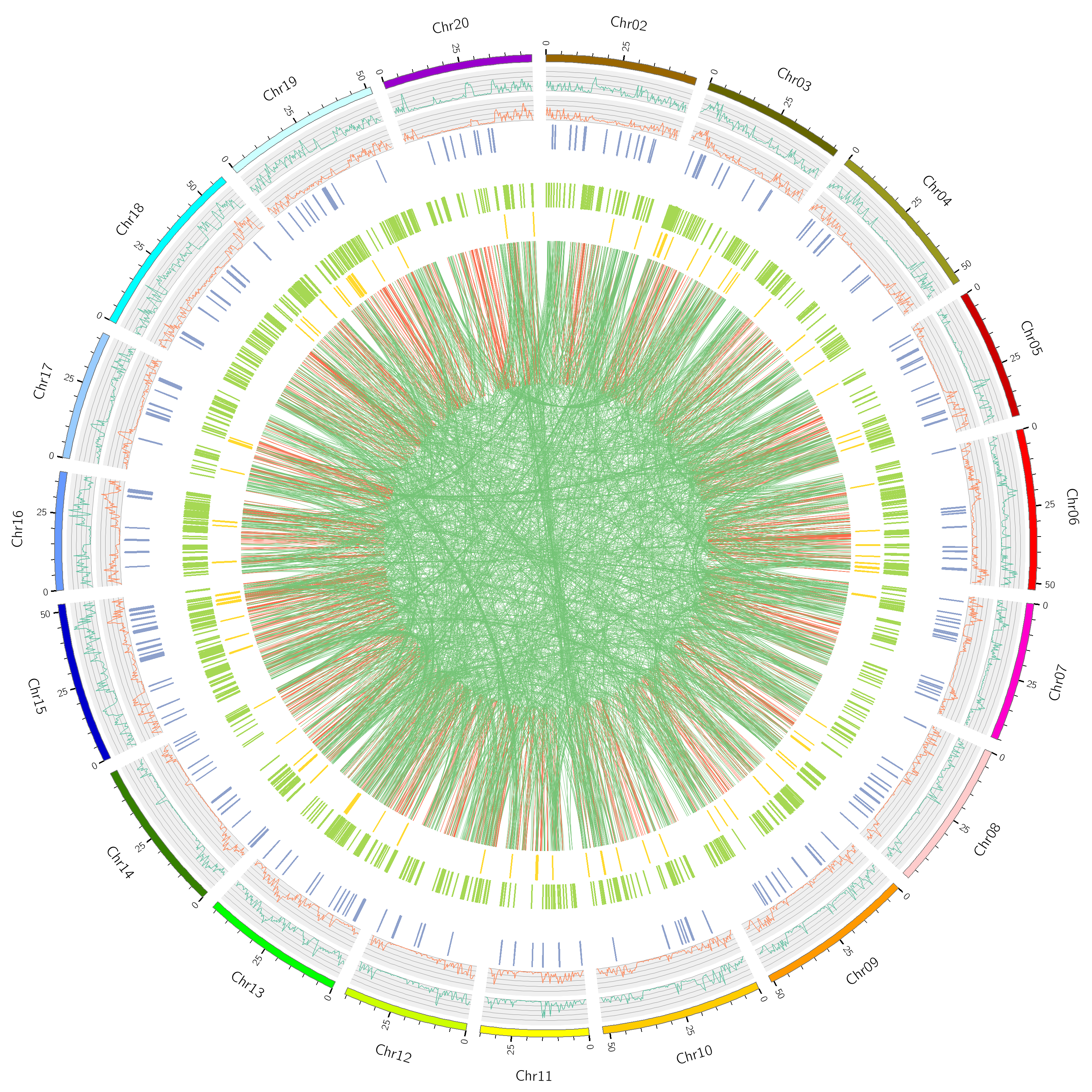

Supplement: Supplementary file 1 [file plants-11-00028-s001.zip › Figure S2. The distribution of the variation of Zhexian No. 9 on the chromosomes.jpg]

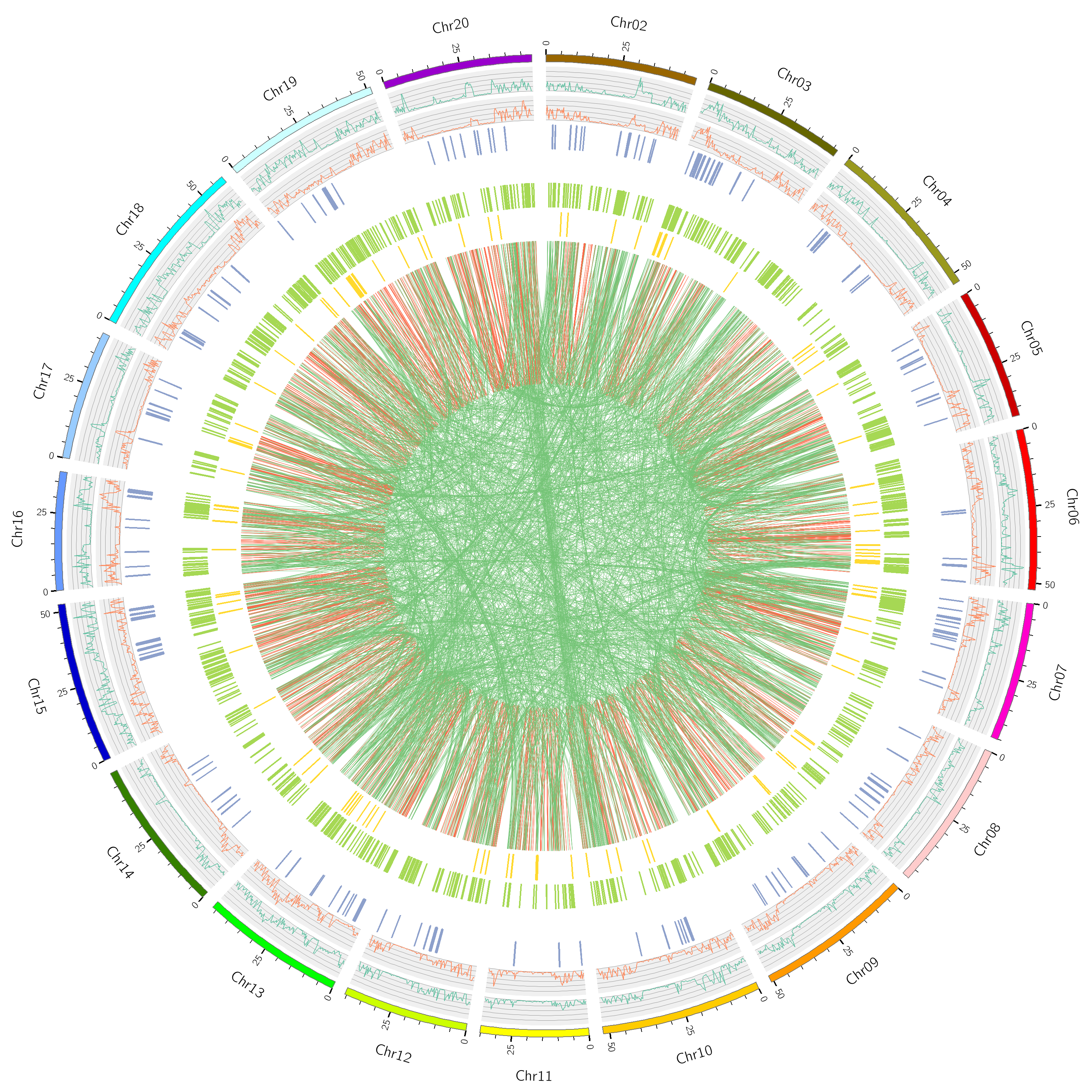

Supplement: Supplementary file 1 [file plants-11-00028-s001.zip › Figure S3. The distribution of the variation of Zhexian No. 10 on the chromosomes.jpg]
